# Evaluating blood oxygen saturation measurements by popular fitness trackers in postoperative patients: A prospective clinical trial

**DOI:** 10.1016/j.isci.2023.108155

**Published:** 2023-10-06

**Authors:** Philipp Helmer, Philipp Rodemers, Sebastian Hottenrott, Robert Leppich, Maja Helwich, Rüdiger Pryss, Peter Kranke, Patrick Meybohm, Bernd E. Winkler, Michael Sammeth

**Affiliations:** 1 Department of Anaesthesiology, Intensive Care, Emergency and Pain Medicine, University Hospital Würzburg, Oberdürrbacher Str. 6, 97080 Würzburg, Germany; 2 Department of Software Engineering, Faculty of Computer Science, University Würzburg, Am Hubland, 97074 Würzburg, Germany; 3 Institute for Clinical Epidemiology and Biometry, University Würzburg, Josef-Schneider-Str. 2, 97080 Würzburg, Germany; 4 Department of Applied Sciences and Health, Coburg University, Friedrich-Streib-Str. 2, 96450 Coburg, Germany

**Keywords:** Health sciences, Clinical measurement in health technology, Bioelectronics

## Abstract

Blood oxygen saturation is an important clinical parameter, especially in postoperative hospitalized patients, monitored in clinical practice by arterial blood gas (ABG) and/or pulse oximetry that both are not suitable for a long-term continuous monitoring of patients during the entire hospital stay, or beyond. Technological advances developed recently for consumer-grade fitness trackers could—at least in theory—help to fill in this gap, but benchmarks on the applicability and accuracy of these technologies in hospitalized patients are currently lacking. We therefore conducted at the postanaesthesia care unit under controlled settings a prospective clinical trial with 201 patients, comparing in total >1,000 oxygen blood saturation measurements by fitness trackers of three brands with the ABG gold standard and with pulse oximetry. Our results suggest that, despite of an overall still tolerable measuring accuracy, comparatively high dropout rates severely limit the possibilities of employing fitness trackers, particularly during the immediate postoperative period of hospitalized patients.

## Highlights

• The accuracy of O2 measurements by fitness trackers is tolerable (RMSE ≲4%)
• Correlation with arterial blood gas measurements is fair to moderate (PCC = [0.46; 0.64])
• Dropout rates of fitness trackers during O2 monitoring are high (∼1/3 values missing)
• Fitness trackers cannot be recommended for O2 measuring during critical monitoring

## Graphical abstract

**Figure:**
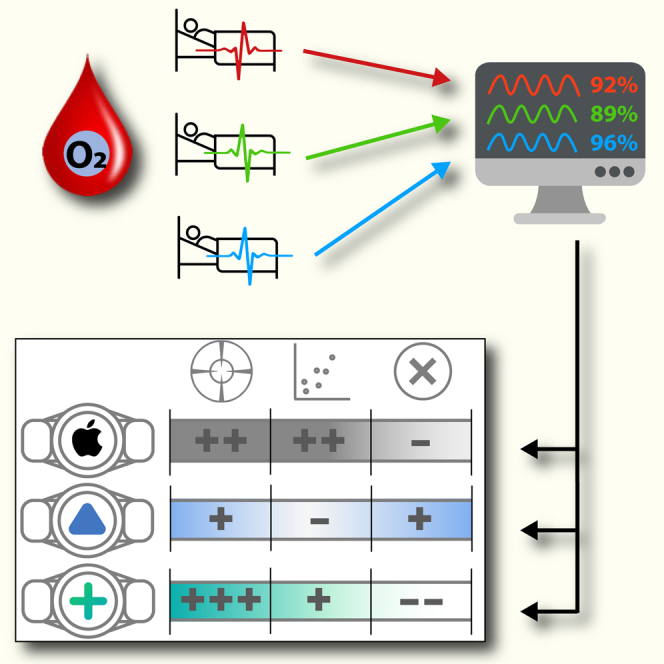

## Introduction

Arterial blood oxygen saturation is the most commonly used surrogate parameter for pulmonary gas exchange, and therefore paramount in many different use cases in modern healthcare. In intensive care medicine and anesthesia, the continuous noninvasive measurement of oxygen saturation constitutes an integral element for more than 30 years. Moreover, tight monitoring of blood oxygen saturation is essential for patients with infectious diseases suffering from silent hypoxemia (e.g., COVID-19),^1^ for patients with chronic diseases (e.g., obstructive sleep apnoea and chronic obstructive pulmonary disease),^2^^,^^3^ or for hospitalized patients with opioid therapy who may develop central apnea.^4^ Particularly in patients undergoing surgical procedures, an early detection and therapy of hypoxia is crucial.

For almost 60 years now, the gold standard of measuring the functional oxygen saturation (*s*O_2_) in the arterial blood (denominated *Sa*O_2_) has been the arterial blood gas (ABG) analysis. Adopting standard hemoglobin oxygenation nomenclature summarized by Blackburn et al.,^5^ *s*O_2_ represents the proportion of oxy-haemoglobin (O_2_Hb) in the functional hemoglobin complement constituted by O_2_Hb and deoxy-haemoglobin (HHb, Figure S1). Based on multiple wavelength analysis, ABG as well as more advanced pulse oximetry devices can additionally discriminate the physiologically rare dys-haemoglobin derivatives (i.e., carboxy-haemoglobin COHb and methaemoglobin MetHb), and thereby provide *fractional* saturation measurements (*F*) for each of the hemoglobin derivatives (Method S1).

However, the ABG method cannot be applied for continuous *s*O_2_ monitoring, because a designated blood sample has to be drawn from the patient for each *Sa*O_2_ measurement. Motivated by these shortcomings, Aoyagi and Kishi developed the transmissive pulse oximetry (TPO) in 1972.^6^^,^^7^

Thus, analyzing light sent by a clip commonly through the fingertip, TPO attempts to determine *s*O_2_ by measurements at the peripheral capillaries, the so-called *peripheral* oxygen saturation (*Sp*O_2_). Hence, *Sa*O_2_ as well as *Sp*O_2_ both aim to determine the functional oxygen saturation in the blood. While TPO successfully enables the continuous monitoring of *s*O_2_, the mobility of patients is still severely impaired by the finger clip—mostly cabled to the measuring device—impacting on the compliance to wear such devices, especially of awake patients.

Since recently, different consumer-grade manufacturers develop so-called *wearables*, predominantly fitness tracking bands and watches, leveraging the continuous monitoring of *Sp*O_2_.^8^ In contrast to TPO, fitness trackers rely on *reflective* pulse oximetry, with the emitting LED and the sensor/photodiode juxtaposed on a wrist-attached unit, making the use of a finger clip obsolete. This allows for increased mobility and comfort, and also can offer new possibilities for hospitalized patients, patients after hospital discharge or outpatients.

So far, some consumer-grade devices have demonstrated acceptable accuracy for the heart rate monitoring in hospitalized patients,^9^ as well as for measuring *Sp*O_2_ over a broad range of oxygen saturation levels in resting healthy subjects (Bias +0.0% LoA [-4.9; 4.9] in hypobaric chambers^10^; Bias +0.98% LoA [-4.66; 6.62] while breathing a hypoxic gas mixture).^11^ Also employing the Apple Watch in outpatients with chronic lung disease suggests a promising measurement accuracy for *Sp*O_2_ (Bias +0.8% LoA [-2.7; 4.1]).^12^

However, to date, the preponderant part of studies assessing devices in their ability to measure *Sp*O_2_ suffers limitations in the clinical translation, because the study protocols (i) either lack ABG references and thus exclusively rely on comparisons between different devices based on reflective oximetry and TPO,^10^ (ii) they include exclusively healthy subjects and no hospitalized patients,^11^ (iii) they suffer from data loss leading to non-interpretable results,^13^ or (iv) they involve potential conflicts of interest by manufacturers.^11^ Therefore, the applicability of fitness trackers in hospitalized patients suffering from multiple diseases, and also the accuracy of *Sp*O_2_ measurements in patients undergoing surgical procedures, remains unclear.

We therefore conceived and conducted a pioneering prospective study to systematically investigate the accuracy of on-demand *Sp*O_2_ measurements by three popular fitness trackers (i.e., the Apple Watch 7, the Garmin Fenix 6 pro, and the Withings ScanWatch), employing a cross-over design in patients after moderate or major surgery. In order to objectively assess subtle differences between the devices, we validated the fitness tracker *Sp*O_2_ measurements thoroughly with clinical gold standard references. To provide an enhanced interpretability and also comparability of our results, we employed as *s*O_2_ reference values in the surgical patients the clinically established methods ABG, providing *Sa*O_2_ measurements, as well as TPO, yielding *Sp*O_2_ readings. Moreover, measurements were collected under controlled conditions, with patients at rest, because even professional devices can be seriously affected by motion artifacts.^14^

## Results

### Overview of the cohort

**Figure 1:**
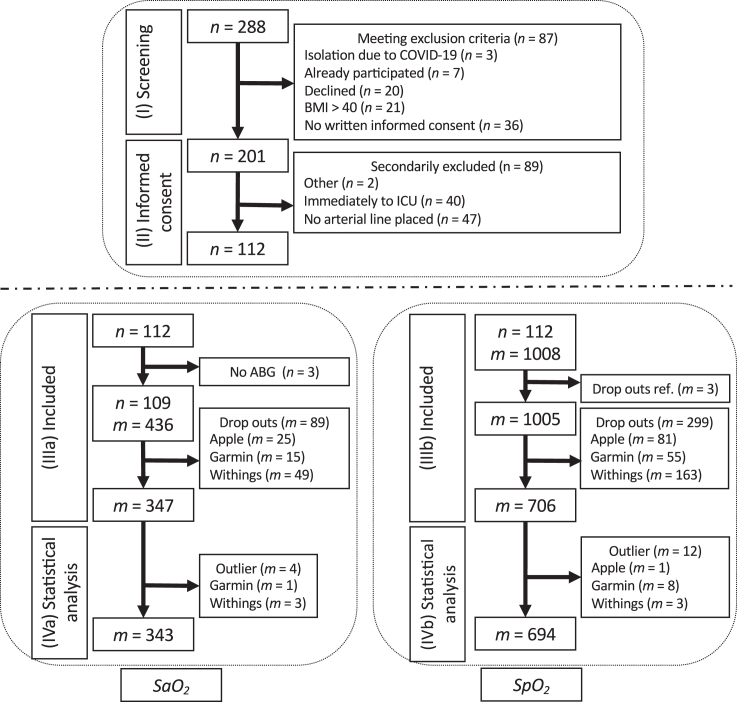
Study flow chart Top : (I) During screening of 288 patients, 87 of these met exclusion criteria. (II) Informed consent was obtained by 201 patients, of whom 112 patients could be included in the measurements. Bottom : For the *Sa*O_2_ benchmark (left ), 347 valid measurements (IIIa) were obtained, which after outlier removal led to 343 measurement pairs to be considered in our statistical analysis (IVa). Regarding the *Sp*O_2_ benchmark, 706 valid measurements (IIIb) yielded 694 pairs to be evaluated. Of note, data acquisition in (IIIa, IVa) and (IIIb, IVb) are based on the same patient cohort recruited in (I, II).

After initial screening of 288 patients, 201 patients gave written informed consent. Of these, 89 patients were secondarily excluded, because they either were transmitted to an ICU immediately after operation or no arterial line was placed during the surgical procedure (Figure 1, top panel). The 112 remaining patients constituted our study cohort, with ages ranging from a minimum of 24 years old (y.o.) to a maximum of 92 y.o. (median 68 y.o., IQR of 16 y.o.). Patients in our study were slightly overweight, with a median BMI of 26.9 kg/m^2^ (IQR 6.2 kg/m^2^). The included patients further predominantly exhibited a Caucasian phenotype, reflected by a median value on the Fitzpatrick Scale of 2 (IQR 1) and by mostly minimal underarm hairiness (median 1 and IQR 2 on our inhouse scale). The median wrist circumference was 18 cm (IQR 2 cm). During routine patient care in the PACU, 45.5% of patients required oxygen supply, with a minimum quantity of 1 L/min O_2_ supplied through nasal cannula and a maximum of 8 L/min O_2_ supplied through a face mask (Table S2).

### Quality control

Figure 1 (bottom left panel) summarizes the number of measurements obtained from the n = 112 patients of the cohort for the *Sa*O_2_ benchmark: no routine ABG was available for 3 of the patients, who consequently could not be considered in the *Sa*O_2_ benchmark. The remaining n = 109 patients were timestamped matched with the corresponding measurements by TPO and by each of the 3 attached fitness trackers (in total *m* = 4 × 109 = 436 measurements), discarding 89 dropouts (Apple: 25, Garmin: 15, Withings: 49) by these devices (20.41% of the attempted measurements). Subsequently we removed in total 4 outliers (0.92%) with a real error of < -9% or >7% (Figure S2), composed by 3 Withings and 1 Garmin measurement, 343 paired measurements could successfully be included in the *Sa*O_2_ benchmark.

In order to assess the accuracy of fitness trackers (Figure 1, bottom right panel), we benchmarked their *Sp*O_2_ readings against the corresponding TPO measurements. To this end, we attempted 3 measurements on each of the 3 devices attached to each of the patients, yielding a total of *m* = 1,008 tracker measurements in the entire cohort (n = 112 patients). We recorded 1 dropout in the TPO reference readings (0.3% of the TPO measurements), reducing the number of comparable tracker readings to *m* = 1,005 (i.e., one measurement for each of the fitness trackers could not be compared). The fitness trackers exhibited 299 (29.75% of all measurements) dropouts in total, with the highest dropout rate 48% in the Withings measurements, followed by 24.2% dropouts in the Apple measurements, and 16.4% dropouts in the Garmin measurements. The remaining *m* = 706 successful tracker *Sp*O_2_ measurements were compared to their corresponding *Sp*O_2_ reference values obtained by TPO, identifying 8 of the Garmin, 3 of the Withings and 1 of the Apple measurements as outliers. Purging our dataset from these 12 outliers (1.19%) left us with *m* = 694 measurements for benchmarking.

### *Sa*O_2_ benchmark

**Figure 2:**
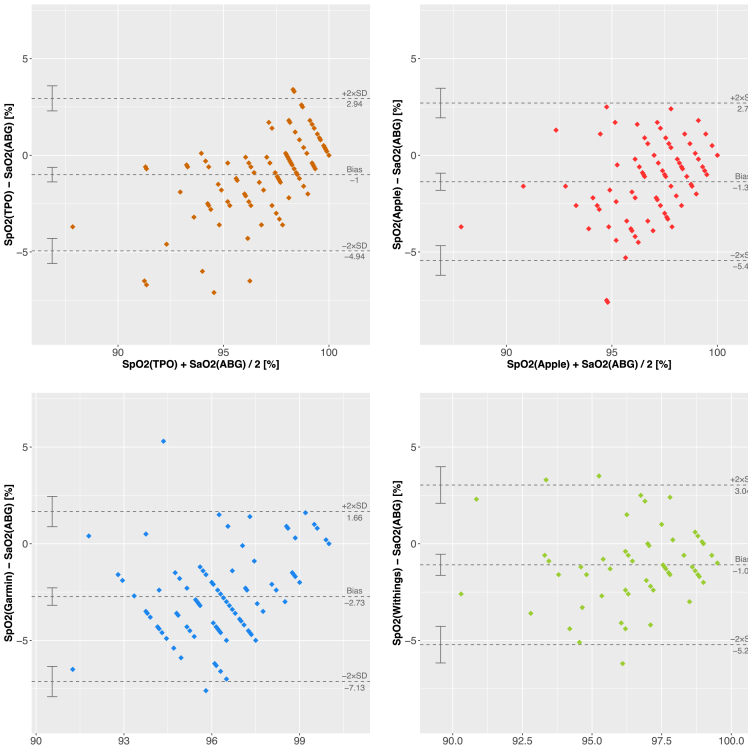
Bland-Altman plots comparing *Sa*O_2_ measurements by ABG to the *Sp*O_2_ readings of each of the investigated devices, including TPO Following the visualization proposed by Bland and Altman, scatterplots showing the real errors of the measurements (y axis: *Sp*O_2_ measurements minus *Sa*O_2_ reference) stratified by the mean of each measurement pair (x axis). Dashed horizontal lines mark the bias (*B*), i.e., the arithmetic average of all real errors with the limits of agreement (LoA) as determined by an offset of ±2 times the standard deviation (*SD*). Error bars show the 95% confidence interval (CI) for the bias and both LoA. For the ease of comparison, data points are color-coded, specifically for each of the devices: TPO = orange (top-left); Apple = red (top-right); Garmin = blue (bottom-left); Withings = green (bottom-right).

All four benchmarked devices underestimated the oxygen saturation by approximately 1%–3% on average, compared to *Sa*O_2_ measurements (Figure 2). The Bland-Altman indicators of TPO, Apple and Withings are relatively close to each other, with TPO exhibiting the smallest bias of −1% and the tightest LoA boundaries (-4.94%; 2.94%). In contrast, Garmin exhibits the highest bias of and also slightly larger variation (−2.73%; LoA [-7.13%; 1.66%]).

**Figure 3:**
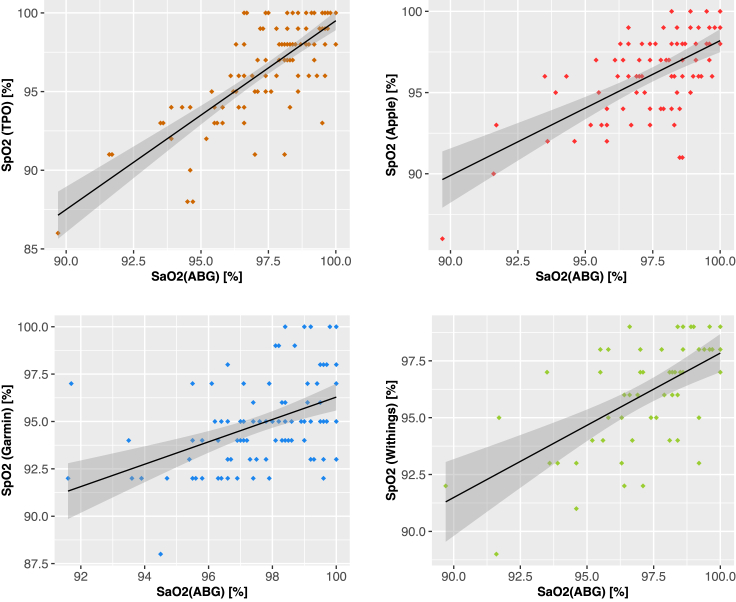
Linear correlation assessment of the blood oxygen saturation measurements comparing the investigated devices to ABG Scatterplots localize each of the paired measurements (*x*,*y*) by the *Sa*O_2_ reference value obtained by ABG (*x*) and the corresponding *Sp*O_2_ measurement of the benchmarked device (*y*). The black solid line depicts the linear regression model, with the 95% confidence interval shaded in gray. Color codes for the devices: TPO = orange (top-left); Apple = red (top-right); Garmin = blue (bottom-left); Withings = green (bottom-right).

Next, we investigated the linear correlation between the *Sa*O_2_ reference and the *Sp*O_2_ readings (Figure 3). Pearson coefficients suggest a high (*r* = 0.78, p < 0.001) correlation between the *Sp*O_2_ values by TPO and the *Sa*O_2_ references, but rather fair (*r* = 0.46, p < 0.001) to moderate (*r* = 0.64, p < 0.001) correlations of *Sp*O_2_ readings by fitness trackers with the *Sa*O_2_ values. These differences in correlation are also reflected by the condensed RMSE indicators, where TPO demonstrates the lowest error with 2.2% (CI [1.83%; 2.64%]) and Garmin with 3.5% (CI [3.18%; 3.88%]). Moreover, linear regression of the paired measurements pinpoints a slope of 1.2 for TPO, whereas all tracker devices yield a slope of < 1 (Apple: 0.83; Garmin: 0.59; Withings: 0.64; Table S3).

### *Sp*O_2_ benchmark

**Figure 4:**
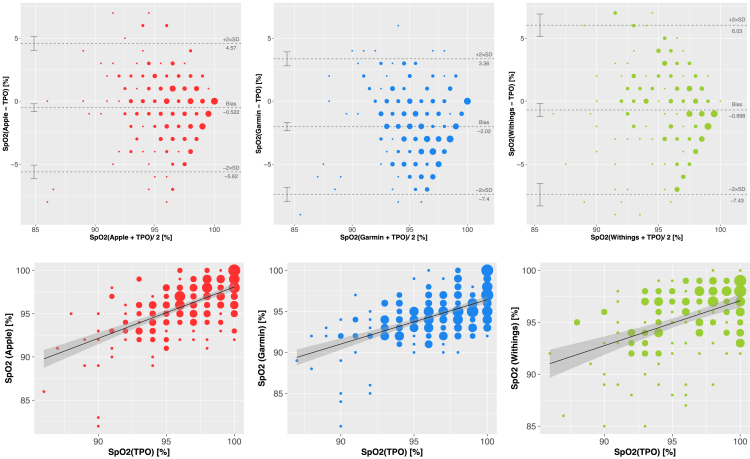
Agreement of *Sp*O_2_ measurements between fitness trackers and TPO Bland-Altman diagrams (upper ) and scatterplots (lower ) assess the agreement between the *Sp*O_2_ readings obtained by fitness trackers to the *Sp*O_2_ reference values defined by TPO measurements. Due to the discrete nature of the *Sp*O_2_ measurements, multiple data points coinciding at the same coordinates are visualized by circles with varying diameters. The black solid line depicts the linear regression model, with the 95% confidence interval shaded in gray. Color codes for the fitness trackers: Apple = red (left); Garmin = blue (center); Withings = green (right).

In accordance with the *Sa*O_2_ benchmarking, the Bland-Altman comparisons show that the *Sp*O_2_ values measured by fitness trackers also underestimate the peripheral oxygen saturation determined by the TPO less (upper panels of Figure 4). The reduced bias is expected, because our previous results already demonstrated that also the *Sp*O_2_ measurements by TPO slightly underestimate *Sa*O_2_ the saturation levels determined by ABG (Figure 2).

In our *Sp*O_2_ Bland-Altman analysis, we observe the best agreement with TPO by Apple (bias: −0.5%; CI [-0.84%;-0.21%]), whereas Garmin still exhibits the largest discrepancy with the reference (bias: −2.02%; CI [-2.34%;-1.70%]). Consistently, Apple *Sp*O_2_ readings also exhibit a very similar correlation with TPO as with ABG measurements (*r* = 0.62 vs. 0.64). In contrast, Pearson coefficients of the Withings *Sp*O_2_ readings strongly differ in both benchmarks (*r* = 0.46 vs. 0.6). However, the *Sp*O_2_ measurements by all fitness trackers score an RMSE < 4% and an MAPE <3%, regardless of their comparison with *Sp*O_2_ (TPO) or with *Sa*O_2_ (ABG) references. Table 1 compares the most important statistical indicators of both benchmarks, and a complete summary is provided in Table S3.

**Table 1:** Comparison between the *Sa*O_2_ and the *Sp*O_2_ benchmarking indicators

	TPO	Apple	Garmin	Withings
*m*	109	84	93	57
	–	253	272	169
*Dropout rate* (%)	0	22.94	13.76	44.95
	–	24.18	16.42	48.66
*RMSE* (%) [CI]	2.20 [1.83; 2.64]	2.44 [2.07; 2.95]	3.50 [3.18; 3.88]	2.32 [1.96; 2.8]
	–	2.60 [2.34; 2.89]	3.36 [2.11; 3.6]	3.43 [3.07; 3.82]
*Bias* [CI]	−1.00 [-1.38;-0.63]	−1.37 [-1.81;-0.93]	−2.73 [-3.18;-2.28]	−1.09 [-1.64;-0.54]
	–	−0.52 [-0.84;-0.21]	−2.02 [-2.34;-1.70]	−0.70 [-1.21;-0.19]
*r*	0.78	0.64	0.46	0.60
	–	0.62	0.56	0.46
*r*_*c*_	0.66	0.53	0.24	0.54
	–	0.61	0.45	0.45

The table summarizes the most relevant indicators of our benchmarks for evaluating the measurement accuracy of the assessed devices (column headers), contrasting the comparisons to *Sa*O_2_ references (ABG, white lines) and with the comparisons to *Sp*O_2_ references (TPO, gray lines). *m* = number of data points. CI, confidence interval; LoA, limits of agreement; *r*, Pearson Correlation coefficient; r_c_, Lin’s concordance coefficient.

### Potential confounders

**Figure 5:**
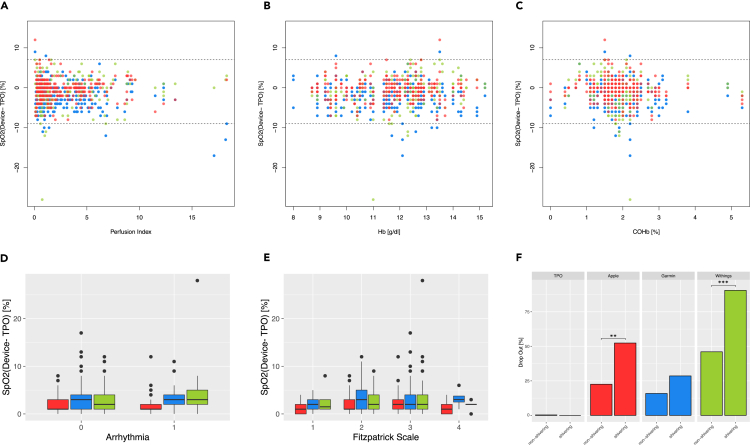
Analysis of potential confounders Patients were segregated in different cohorts according to their attributes classified by variables of different nature (x axis), to assess potential influences on the fitness trackers readings (y axis). In all diagrams, the colors identify the device: TPO = orange; Apple = red; Garmin = blue; Withings = green. (A) the real errors are stratified by perfusion index. (B and C) characteristics of the ABG analysis. (D and E) boxplot visualisations of the absolute errors binned by categorical classifications of the patient attributes. (F) barplots contrasting the dropout rate in non-vs. shivering patients after surgery. ∗∗p < 0.01; ∗∗∗p < 0.001 (Fisher’s Exact Test).

Our Bland-Altman analyses already confirmed that the real errors of the tracker *Sp*O_2_ readings are generally not correlated to particularly high or low blood oxygen saturations of the patient. We therefore conducted an exhaustive investigation on influences by potential confounders in sub-cohorts of our study on the measurement accuracy of fitness trackers. To this end, we segregated the patients in our study according to the recorded perfusion index (PI), the concentration of total hemoglobin (Hb), the fractional saturation of carboxy-hemoglobin (*F*COHb, Figure S1) and of met-haemoglobin (*F*MetHb, Figure S1), BMI, body height, weight, wrist circumference, the ASA Score, skin tonality (Fitzpatrick Scale), degree of hairiness on the forearm, the presence of arrhythmia as well as postoperative shivering of the patients (Figures 5 and S3).

In a nutshell, none of the variables assessed in Figure 5 exhibited a coherent or, respectively, significant impact on the measurement accuracy of the examined devices. However, careful analysis revealed that dropouts by fitness trackers accumulate particularly in the cohort of patients with postoperative shivering: whereas the TPO measurements are not affected in this cohort, each of the tracker devices shows a higher proportion of dropouts in shivering patients (Table S4). These differences in the dropout rate are highly significant for the Apple (p.value < 0.01) and the Withings (p.value < 0.001), and also present in Garmin measurements (16% vs. 29%, Figure 5F).

## Discussion

The objective of our study was to evaluate the accuracy of *Sp*O_2_ oxygen saturation measurements yielded by consumer-grade fitness trackers. To this end, we compared the obtained *Sp*O_2_ estimates with the clinical gold standard for measuring *Sa*O_2_ by ABG analyses and for measuring *Sp*O_2_ by TPO. Based on the thresholds by ISO Standard 80601-2-61:2019,^15^ an accuracy of RMSE ≤ 4% is required for “basic safety and essential performance of pulse oximeter equipment.” Considering exclusively the successful measurements, all of the investigated tracker devices comply with these limits within the range of 90%–100% *Sp*O_2_. However, with the observed dropout rates of ∼30% on average, consumer-grade fitness trackers fail by two orders of magnitude more frequently than standard TPO (0.3% dropout rate) to obtain *Sp*O_2_ readings. In our study, the dropout rates also varied by a factor of about 3 between different models (Withings 49%, Apple 24%, Garmin 16%).

At the same time, the cumulative error measures (e.g., MAE and RMSE) are increasing in the ranking Withings ≺ Apple ≺ Garmin (Tables 1 and S3); i.e., the number of successful *Sp*O_2_ measurements inversely correlates to their observed accuracy regarding *Sa*O_2_ reference values. Under the hypothesis that dropouts are caused by insufficient sensor capabilities, we would expect higher measurement errors to correlate with higher dropout rates. Therefore, our observations on the competitiveness between error and dropout suggest differences in the stringency of internal quality control algorithms of each of the benchmarked trackers. These considerations are also supported by indicators from our correlation analyses, yielding lower Pearson Correlation for Garmin as compared to Apple and Withings (0.46 vs. 0.64 and 0.6) as well as lower Lin’s Concordance (0.24 vs. 0.53 and 0.54) coefficients (Figure 3; Table S3). Our results are in line with the study of Schiefer et al. investigating the Garmin Fenix 5X Plus in 13 healthy volunteers (MAPE 9.77%; mean *Sp*O_2_ difference 7.0%).^16^

Moreover, we observed that the *Sp*O_2_ readings by all pulse oximetry devices are coherently underestimating the *Sa*O_2_ reference, with average biases of approximately (−1%) to (−3%) (Figure 2; Table 1). It has been reported that, albeit differences in absorption spectra, pulse oximetry can missense COHb as O_2_Hb, leading to an overestimation of *Sp*O_2_ measurements.^17^^,^^18^ However, these effects have been demonstrated negligible by the laws of physics (i.e., the Beer-Lambert Law) and also by corresponding *in vitro* experiments for *F*COHb saturations of up to 20%.^19^ In our patient cohort, ABG analysis indicated a median/mean *F*COHb of 1.9% (IQR [1.6%; 2.2%], maximum at 5.3%), debunking potential COHb biases. Of note, the TPO sensor employed in our study (Philips M1191B) exhibits negative biases also in the reference benchmarks by the manufacturer (personal communication). Moreover, in our study, the accuracy of RMSE 2.2% for TPO is in agreement with an ABG benchmark of two comparable clinical standard pulse oximeters; i.e., the Massimo Radical (RMSE 3.95%) and the Nellcor N-600 (RMSE 2.1%), across a comparable range of sO_2_ saturations [90%; 100%].^20^

When considering the *Sp*O_2_ values yielded by TPO as a reference, the observed biases decrease for each of the tracker devices (Figure 4; Table S3), as expected by also TPO measurements slightly underestimating the ABG measurements (Figure 2). Notwithstanding these similarities, the spread of the error (i.e., LoA) between tracker *Sp*O_2_ estimates and TPO *Sp*O_2_ values increase as compared to the *Sa*O_2_ benchmark, indicating the presence of random and therefore independent variation in the *Sp*O_2_ measurements of each device. Overall, in our *Sp*O_2_ benchmark, Apple exhibits the lowest RMSE (2.60%) as compared to Garmin (3.36%) and Withings (3.43%). In comparison to our previous *Sa*O_2_ benchmark, Apple also improves the concordance (*r*_*c*_) while maintaining the linear correlation (*r*), but does not achieve the high correlation coefficient (*r* = 0.995) reported by a previous study comparing *Sp*O_2_ readings by the Apple Watch 6 to commercial pulse oximeters in patients with interstitial lung disease and COPD.^12^ However, we observe an overall rather moderate to poor correlation (*r*≲ 0.6) between the trackers and the clinical TPO standard (Table 1).

Most of the potential confounders we analyzed exhibited no significant and clinically relevant impact on the measurement accuracy of the investigated devices (Figures 5A–5E). However, the dropout rates are significantly increased in patients with postoperative shivering (Figure 5F). These observations further support the previously formulated hypothesis that dropout is governed—at least in part—by internal quality control cut-offs.

In summary, our results suggest that fitness trackers *Sp*O_2_ readings based on reflective pulse oximetry are less accurate and substantially more prone to increased dropout rates compared to the clinically established TPO. Our results are supported by a previous study that TPO succeeded in detecting hypoxemia, whereas reflective wrist-worn devices had to be excluded from analysis due to *Sp*O_2_ estimation performance issues.^13^ One rationale behind these observations is that the signal-to-noise ratio is much lower for reflective compared to transmissive pulse oximetry, with readings hampered by motion artifacts, reduced perfusion, stronger interferences by tissue, and a higher exposure to external light.^11^ Therefore, it is not surprising that similar dropout rates (26% ± 24%) were reported also for a reflective pulse oximeter attached to the chest (SmartCardia).^20^

### Conclusion

In our cohort, all of the investigated devices achieved an RMSE ≤ 4% for the measurement of *Sp*O_2_, thereby complying with the threshold of the ISO standards for medical-grade reflective pulse oximeters. However, the fair to moderate correlations of the investigated devices with the clinical gold standard, and importantly their high dropout rates of up to 50%, render an implementation of fitness trackers in the postoperative clinical setting challenging and limited to constrained use cases. Based on our results, a wide scale implementation of fitness trackers for the continuous monitoring of blood oxygen saturation in postanaesthesia clinical routine for the reliable detection of hypoxia cannot be recommended at this stage.

### Limitations of the study

Our study is not free of limitations. The study protocol does not fulfill the standards of ISO norm (80601-2-61:2019) requiring at least 200 ABGs equally balanced in the range of 70–100%.^15^ In our cohort, 103 samples had oxygen saturations between 90 and 95%, and only 9 samples showed hypoxia as defined by *Sp*O_2_ < 90%. Due to obvious considerations about the potential harm of patients, it is not possible to induce hypoxia in our investigated collective of postoperative, diseased patients. In this regard, our trial has not been designed as a certification study from the beginning.

In principle, the reliability of measurements integrates two compounds—the measurement accuracy and its reproducibility. Since in our study only three measurements per patient and device were collected, our possibilities to draw conclusions on the reproducibility of the observed accuracy are limited. Regarding our evaluations of the measurement accuracy, we synchronized the measurements on the benchmarked devices closely with the routine collection of ABG samples, but a time shift of ≤ 30s between the two interrogations could technically not be excluded. As a further aggravating factor, the time intervals for determining *Sp*O_2_ also vary among the benchmarked devices, and even among their single measurements. These variations in the time intervals of measuring cannot be modified and also are not consistently specified by the manufacturers.

The medical transmissive pulse oximeter, we employed averages the blood oxygen saturation over the last 3–6s,^21^ whereas Apple over approximately 15s,^22^ Withings over 30s,^11^ and Garmin according to our experiences exhibits highly fluctuating measurement intervals. Also, an additional delay between the end of the actual sampling interval and the time point when the result is displayed on an investigated device cannot be excluded. It is reassuring that our observations on the measurement accuracy of TPO are in line with the results of a multicentre study reporting comparable deviations in the Bland-Altman analysis (Bias −1.2% vs. −1%).^23^ Therefore, potential biases caused by different sources of variability in the sampling intervals of AGB and TPO seem to play a subordinate role in our study.

## STAR★Methods

### Key resources table

**Table:** 

REAGENT or RESOURCE	SOURCE	IDENTIFIER
**Biological samples**		

Arterial Blood	This manuscript	N/A

**Software and algorithms**		

R Project for Statistical Computing	Dessau and Pipper^27^	RRID:SCR_001905
ggplot2 package	Wickham^28^	RRID:SCR_014601
DescTool package	Signorell^30^	N/A
Rcompanion package	Mangiafico^31^	N/A

**Other**		

Philips Healthcare: Intellivue X3, MX750, M1191B	Philips Healthcare Inc. Andover/MA, USA	RRID:SCR_00865
GEM 5000 Premier BGA System	Werfen GmbH, Munich, Germany	N/A
Apple Watch 7	Apple Inc. Cupertino/CA, USA	N/A
Garmin Fenix 6 pro	Garmin Ltd. Olathe/KS, USA	N/A
Withings ScanWatch	Issy-les-Moulineaux, France	N/A

### Resource availability

#### Lead contact

Further information and requests for resources should be directed to the corresponding author contact, Dr. Helmer Philipp (helmer_p@ukw.de).

#### Materials availability

This study did not generate new unique reagents.

### Experimental model and subject details

#### Study conducts and ethics

This prospective validation study was performed between November 2021 and May 2022 at the Department of Anaesthesiology, Intensive Care, Emergency and Pain Medicine at the University Hospital Würzburg, Germany. Approval of the study protocol was obtained from the ethics committee of the University of Wuerzburg, Germany (ref. no. 145/21_c). The study was conducted in accordance with the good clinical practice guidelines, the declaration of Helsinki (2013, Fortaleza) as well as the guidelines for wrist worn consumer wearables.^24^ Written informed consent was obtained from all study participants prior to surgical procedures. Study protocol of the “Monitor trial” was registered on clinicaltrials.gov (accession no. NCT05418881) and the results of *Sp*O_2_ measurements are presented in this article. The study was designed, conducted and analyzed without financial support or any contribution of industrial partners to avoid potential conflicts of interest.

#### Study design and population

We screened patients (≥18 y.o.) scheduled for elective moderate or major surgery, according to ESC/ESA Guidelines,^25^ with the expected requirement of an arterial line being placed for continuous invasive blood pressure monitoring during the surgical procedure, but without a postoperative invasive ventilation being anticipated. Primarily excluded outpatients were constituted by critically ill (i.e., ASA V) patients, obese patients (body-mass-index >40 kg/m^2^), and also patients with infectious diseases to ensure hygienical safety. Furthermore, patients who were unable to provide written informed consent respectively who could not understand/read the patient information sheet in German language, as well as patients that already had participated in this study before, were excluded. Finally, known allergies to latex, silicone or nickel and extensive pathological skin lesions were considered as contraindications for study participation. Patients without arterial line placed in the course of the surgical procedure, or who were postoperative sedated, ventilated, temporally critically ill or unexpectedly admitted to an intensive care unit immediately were secondarily excluded.

As our study focuses on commercial fitness trackers, we initially screened such devices for their ability to measure *Sp*O2 (Figure S1). From these, we selected the brands Apple, Garmin and Withings based on their popularity in related literature in the field of heath applications. Finally, we selected the correspondingly most advanced model of each of these manufacturers that was commercially available by the time we started our study. Our study investigated three consumer-grade fitness trackers, (i) the Apple Watch 7, (ii) the Garmin Fenix 6 pro, and (iii) the Withings ScanWatch. As our benchmark comprises exclusively the specified model of each brand, we employ the manufacturer’s name as a shorthand abbreviation for each model in our comparison. Before the beginning of our study, anonymised user accounts were set up at the online platform of each manufacturer. After the primary setup and updating the firmware of each device to the latest version to date (Table S1). Subsequently, we employed the devices exclusively offline in our study, in order to prevent any automatic firmware updates with possible changes to the algorithms, which have been demonstrated to be able to affect benchmark results.^26^ To further avoid investigator-based biases, the same two trained and experienced sub-investigators carried out the necessary procedures during the entire study period.

#### Study endpoints/outcome measures

The primary endpoint was defined as the accuracy of the consumer-grade fitness trackers to measure *Sp*O_2_ when compared to the functional oxygen saturation (*s*O_2_, Method S1) defined by ABG (*Sa*O_2_). According to ISO 80601-2-61:2019,^15^ a root-mean-square error (RMSE) ≤4% was defined as a threshold for acceptable accuracy. The secondary endpoints were defined as the measurement accuracy of the investigated devices against TPO, and the analysis of possible confounders biasing systematically the measurements or increasing dropout rates when measuring *Sp*O_2_ by the investigated devices.

### Method details

#### Sample collection

Standard attributes were collected for each of the Caucasian participants, including sex (43 female and 69 male), age, height, weight, BMI, wrist circumference, arrhythmia, skin tonality on the Fitzpatrick’s scale, as well as ASA classification of the patient (Table S2). Additionally, we categorised the hairiness on the forearm by an inhouse developed 4-level scale, with 0 = no forearm hair, 1 = minimal ∼, 2 = moderate ∼, and 3 = extensive hair density on the forearm. Over the time of measuring, the physical activity of study participants, and oxygen flow rate -if oxygen supplement therapy was applied-were documented.

On each of the benchmarked tracker devices, the on-demand *Sp*O_2_ measurements were carried out manually by our two research staff members. The time and the value of each readout was recorded, and simultaneously the *Sp*O_2_ values correspondingly obtained through TPO were copied from the display of the bedside monitors. These manually recorded time points allowed to match the *Sp*O_2_ measurements *a posteriori* to the sampling timestamp of the ABG measurements. If a tracker device failed to determine a *Sp*O_2_ value for the requested measurement, we marked the corresponding reading as dropout.

#### Monitoring vital parameters

During the postoperative observation at the postanaesthesia care unit (PACU), participants were continuously monitored according to clinical standard operating procedures, using IntelliVue X3 (Philips Healthcare, Eindhoven, Netherlands) to display vital parameters on a patient monitor (MX750, Philips Healthcare, Eindhoven, Netherlands). Based on this platform, TPO (FAST Sensor M1191B, Philips Healthcare, Eindhoven, Netherlands), 3-lead electrocardiography (ECG), continuous arterial blood pressure (cABP) measurements as well as cuff-based, non-invasive blood pressure monitoring were employed. Furthermore, as part of the clinical routine procedure, at least one arterial blood gas (ABG) sample was drawn (Blood Gas Sampling System, Werfen, Munich, Germany) via the placed 20G arterial line catheter (*Arrow*, Teleflex Medical, Wayne, Pennsylvania, USA or *Insyte-W*, BD Medical, Franklin Lakes, New Jersey, USA). All ABG samples were analyzed (GEM 5000 Premier, Instrumentation Laboratory Comp. (Werfen), Bedford, USA) immediately after collection.

Each study participant was equipped with three fitness trackers, one of each of the investigated models, which were attached to their wrists by our trained research staff, according to the manufacturer’s instructions. Subsequently, three on-demand *Sp*O_2_ measurements were carried out on each of the devices during the respective patient’s stay at the postanaesthesia care unit. It was ensured that continuously taken TPO *Sp*O_2_ readings remained stable for at least 30 s prior to each on-demand measurement. To avoid potentially confounding factors while measuring, the supplementary oxygen flow rate, the breathing commands and the patient’s body position were kept unchanged during the measuring time interval. The ABG drawn for clinical routine was synchronised in coordination with responsible anesthesia nurses to coincide with the measuring time interval of the investigated fitness tracker, to ensure the comparability of the obtained *s*O_2_ values (Figure S1).

### Quantification and statistical analysis

#### Number of patients per analysis

After initial screening of 288 patients, 201 patients gave written informed consent. Of these, 89 patients were secondarily excluded, because they either were transmitted to an ICU immediately after operation or no arterial line was placed during the surgical procedure (Figure 1, top panel). The 112 remaining patients constituted our study cohort.

#### Assessment of correlation, concordance and dropout

For our data analyses, we employed the statistics platform R (v4.2.0),^27^ employing the ggplot2 (v3.3.6) package for basic visualisations of the data by box, scatter and bar diagrams.^28^ Standard indicators (i.e., the arithmetic mean, the median, the quartiles and the interquartile range IQR) of a single sample were computed by the built-in R function *summary*().^27^ Outliers were defined as data points beyond the whisker limits of a standard boxplot (i.e., ≤1.5x IQR). Correlation and linear regression analyses were performed by the R functions *cor.test*() and respectively *lm*(), and Lin’s Concordance Coefficient was computed with the *CCC*() implementation,^29^ employing the DescTool package.^30^ The significance of variation in the dropout ratio between patient (sub-)cohorts (e.g., non-vs. shivering patients, Figure 5F) was assessed employing Fisher’s Exact Test for count data, as implemented by the *fisher.test*() function.^27^

#### Error measurements

Considering paired measurements (*p*,*q*) composed by an predicted (evaluated) measurement *p* and a reference value *r* (gold standard), we distinguish the *real error* (*p* – *q*), the *absolute (unsigned) error* |*p* – *q*|, and the *percentage error* (|*p* – *q*| ✕ 100/q). Naturally, *real errors* are mirrored to exclusively positive values when considering absolute errors. From these, we compute as cumulative indicators of the error rates the *mean absolute error* (MAE), the *mean percentage error* (MAPE), the *mean squared error* (MSE) and the associated *root-mean-square error* (RMSE), denoted *A*_rms_ in ISO 80601-2-61 (Methods S2).^15^ In order to obtain the 95% confidence interval for an RMSE predictor, we employed bootstrapping as implemented by the *boot*() function of the boot package, employing Efron’s R2 model for pseudo-randomised values with RMSE statistics as implemented by the *efronRSquared*() function in the rcompanion R package,^31^ generating a 50,000 *in silico* replicates of each sample.

#### Bland-Altman analysis

Following the analysis proposed by Bland and Altman,^32^ scatterplots were employed to segregate for every (*p*,*q*) measurement pair the arithmetic average of the predicted and reference values (*p* + *q*)/2 by the inherent real error (*p* – *q*). In these plots, the *bias B* of the benchmarked measurements was estimated by the arithmetic average of all these real errors. The upper and lower limit of agreement (*LoA*) were obtained by an offset of twice the corrected sample standard deviation (i.e., precision *P* in the language of ISO 80601-2-61) of all real errors (Methods S2). For all indicators B and LoA, confidence intervals were determined computationally, assuming a Student’s *t*-distribution model and employing the R function *qt*().

### Additional resources

Additional resources are provided in the supplementary information.

## Data Availability

• The patient data reported in this study cannot be deposited in a public repository in order to preserve patient privacy and confidentiality. • This study did not generate new original code, the sources of the datasets supporting the current study are presented in the “key resources table” and “STAR methods” sections. • Additional information required to reanalyze the data reported in this paper or reproduce the results is available from the lead contact upon request.
