# Supplementary material for: Evaluating blood oxygen saturation measurements by popular fitness trackers in postoperative patients: A prospective clinical trial
Source: iScience. 2023 Oct 6;26(11):108155. doi: 10.1016/j.isci.2023.108155 (PMC10590865; doi:10.1016/j.isci.2023.108155)
Supplement: Document S1. Figures S1–S3, Tables S1–S4, and Methods S1 and S2 [file mmc1.pdf]

iScience, Volume *26*

Supplemental information

Evaluating blood oxygen saturation measurements by popular fitness trackers in postoperative patients: A prospective clinical trial

Philipp Helmer, Philipp Rodemers, Sebastian Hottenrott, Robert Leppich, Maja Helwich, Rüdiger Pryss, Peter Kranke, Patrick Meybohm, Bernd E. Winkler, and Michael Sammeth

Supplementary Information

Evaluating blood oxygen saturation measurements by popular fitness trackers in postoperative patients: a prospective clinical trial

Philipp Helmer, Philipp Rodemers, Sebastian Hottenrott, Robert Leppich, Maja Helwich, Rüdiger Pryss, Peter Kranke, Patrick Meybohm, Bernd E. Winkler, Michael Sammeth

Supplementary Methods

Method S1: Saturation measures of haemoglobin derivatives, related to STAR Methods.

For measuring the O_2 saturation of arterial blood, the results of our ABG analysis comprise for each sample:

the *fractional* O_2 saturation $$FO_2Hb = \frac{[O_2Hb]}{[O_2Hb] + [HHb] + [COHb] + [MetHb]}, \quad (1)$$

and

the *functional* O_2 saturation $$sO_2 = \frac{[O_2Hb]}{[O_2Hb] + [HHb]}. \quad (2)$$

where square brackets indicate the concentration of each of the O_2Hb derivatives. In addition, the ABG output also provides the fractional saturations of the other Hb derivatives (*F*HHb, *F*COHb and *F*MetHb), as determined by substituting [O_2Hb] with the concentration of the corresponding Hb derivative in Equation (1). Note that Equation (1) and Equation (2) imply that the quotient (*F*O_2Hb / *s*O_2) provides (*F*O_2Hb + *F*HHb), the fractional saturation of "effective" haemoglobin derivatives.

Method S2: Error, bias and variance indicator, related to STAR Methods.

Considering the paired datasets of reference (i.e. gold-standard) values $R = \{r_1, r_2, \ldots, r_n\}$ and estimated (i.e. benchmarked) measurements $S = \{s_1, s_2, \ldots, s_n\}$, we compute the so called *mean absolute error* (MAE) by:

$$MAE(R, S) = \sum_{i=1}^{n} \frac{|r_i - s_i|}{n}. \quad (3)$$

Similarly, the arithmetic *mean absolute percentage error* (MAPE) is obtained by:

$$MAPE(R, S) = \sum_{i=1}^{n} \frac{100 \times |r_i - s_i|}{r_i \times n}. \quad (4)$$

The *mean of squared errors* (MSE) is computed equivalently to the residuals used in regression analysis:

$$MSE(R,S) = \sum_{i=1}^{n} \frac{(r_i - s_i)^2}{n} \tag{5}$$

The square root of the MSE indicator further accounts for differences in models applied to the same dataset, derivating the *root mean square error* (RMSE, denoted as A_{rms})[1] estimator by $RMSE(R,S) = \sqrt{MSE(R,S)}$.

Furthermore, we determine the bias *B* between reference measurements *R* and estimator values *R* by the arithmetic average over all real errors.

$$B(R,S) = \sum_{i=1}^{n} \frac{r_i - s_i}{n} \tag{6}$$

Following the nomenclature of Bland and Altman, the upper and lower limits of agreement (*LoA*) are reported by offsets from *B*(*R*,*S*) that correspond to twice the (sample corrected) standard deviation SD of all real errors, alternatively denoted by precision *P*.[15]

$$LoA(R,S) = B(R,S) \pm 2SD \tag{7}$$

with

$$SD = \sqrt{\frac{(r_i - s_i - B(R,S))^2}{n-1}}. \tag{8}$$

Supplementary Items

Supplementary Figures

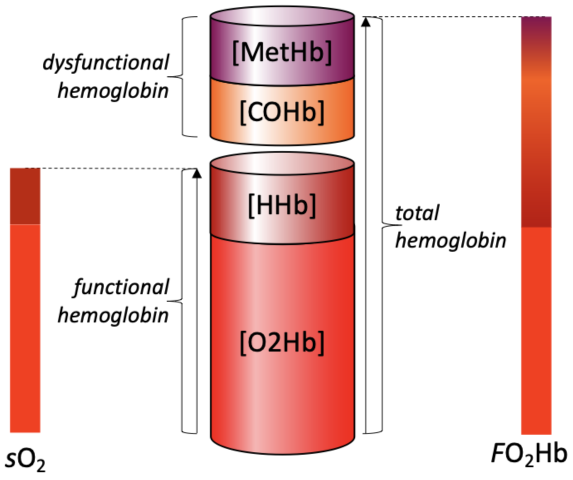

Figure S1: Schema of haemoglobin derivatives, related to STAR-Methods.
Schematic segregation of the *total haemoglobin* content in the blood into a complement constituted by *dysfunctional haemoglobin* derivatives ("dys-haemoglobins"), i.e. haemoglobin molecules that cannot carry oxygen molecules, and into a complement of *functional haemoglobin* molecules that either can bind or are currently bound to O_2 molecules. Dyshaemoglobins comprise carboxy-haemoglobin (COHb) and met-haemoglobin (MetHb), whereas functional haemoglobin is constituted by oxy-haemoglobin (O_2Hb) and deoxy-haemoglobin (HHb).

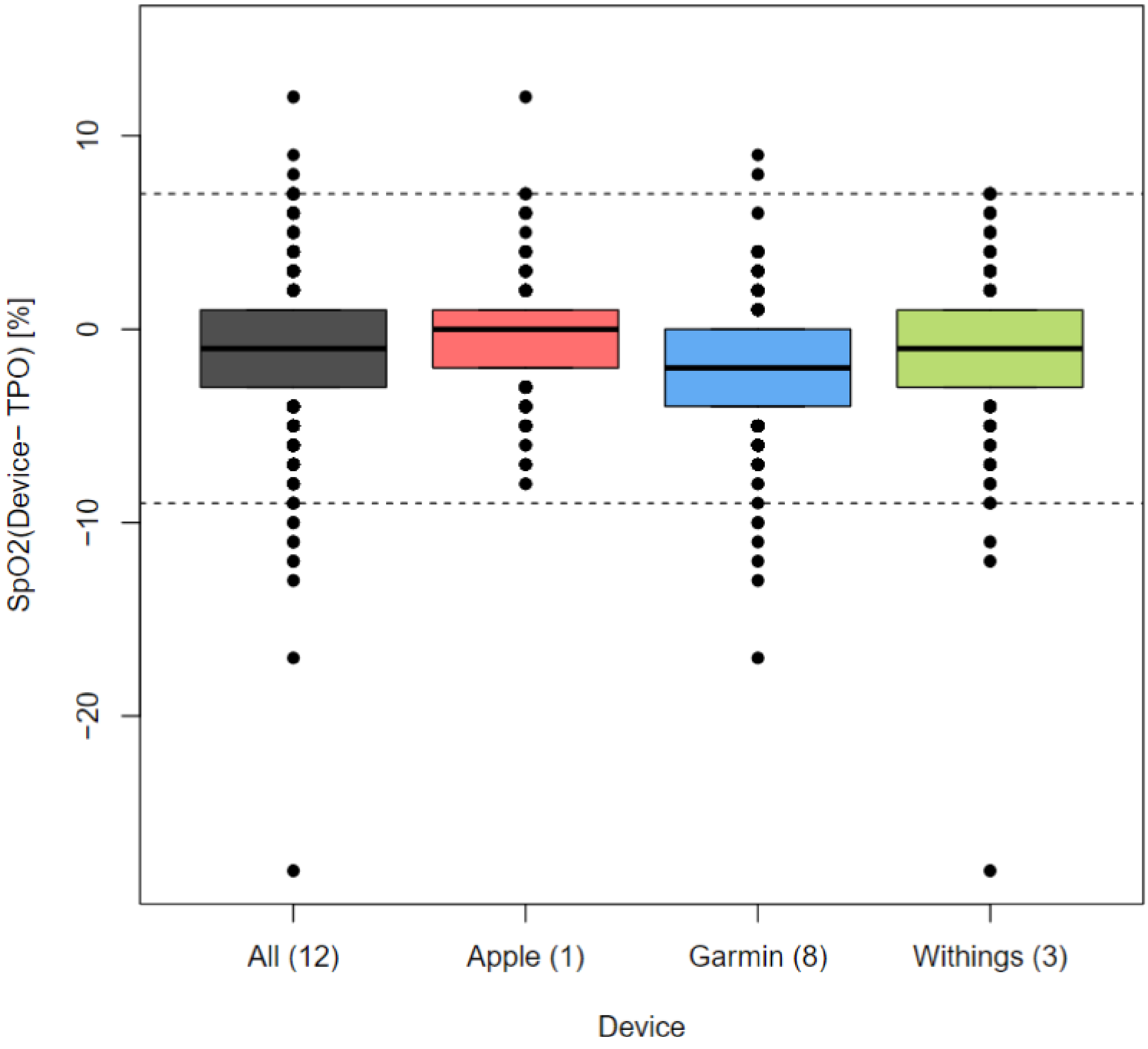

Figure S2: Outlier identification, related to Figure 1.
Outlier distribution depending on the different manufacturers. The boxplots show the distribution of the measured values. The dashed lines indicate the range above which measured values were defined as outliers. A total of 12 measurements were defined as outliers, with these divided between Garmin (8), Withings (3) and Apple (1).

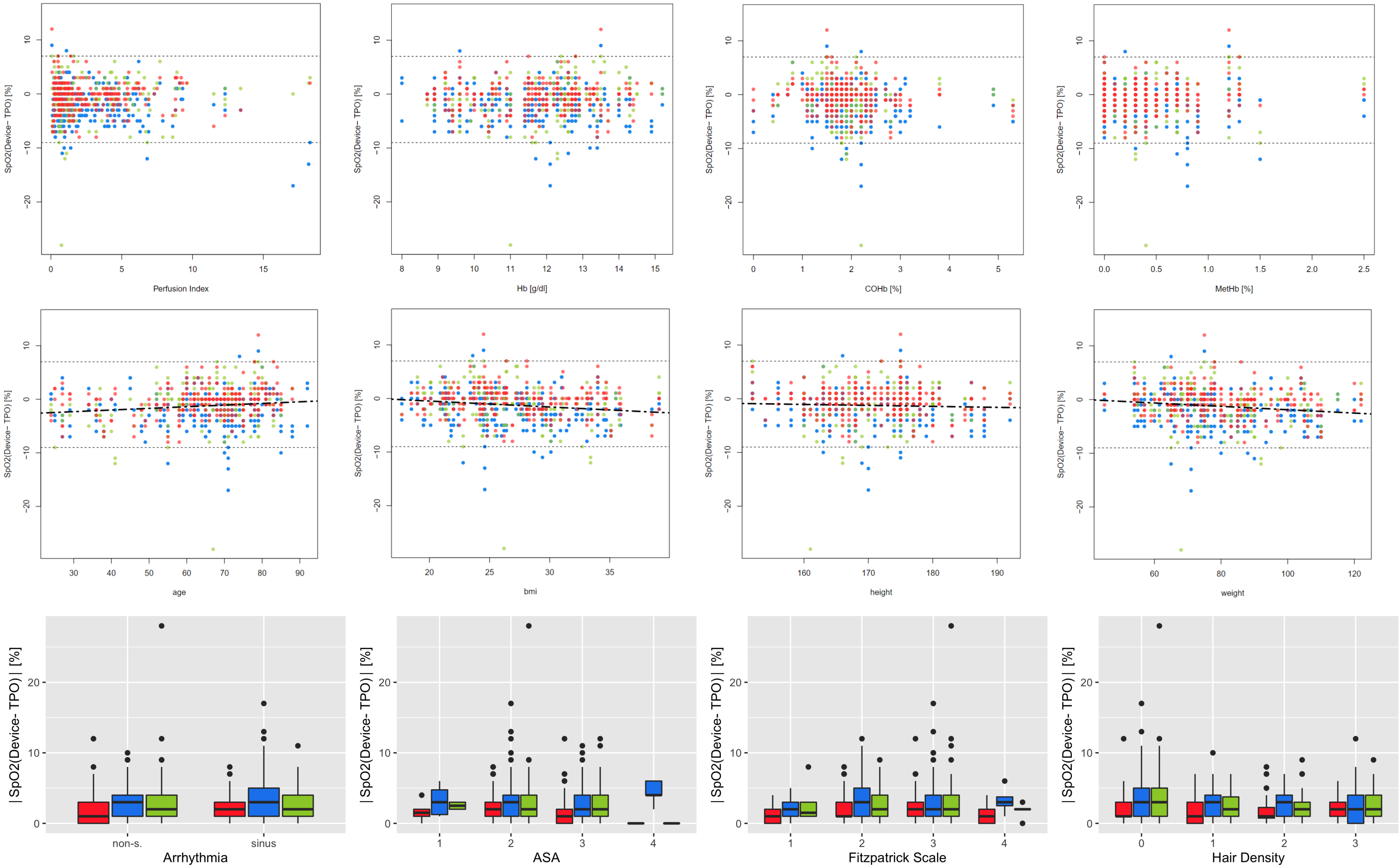
SpO2(Device– TPO) [%]
Perfusion Index
Hb [g/dl]
COHb [%]
MetHb [%]
age
bmi
height
weight
|SpO2(Device- TPO)| [%]
non-s.
sinus
Arrhythmia
ASA
Fitzpatrick Scale
Hair Density

Figure S3. Confounder analysis, related to Figure 5.

Patients were segregated in different cohorts (panels) according to their attributes classified by variables of different nature (x-axis), to assess potential influences (y-axis) on the error. Upper panels: real errors are stratified by characteristics of the blood composition. Middle panels: real errors stratified by physical attributes of the patients. Bottom panels: boxplot visualisations of the real errors binned by categorical classifications of the patient attributes. In all diagrams, the colours identify the device: TPO = orange; Apple = red; Garmin = blue ; Withings = green.

Supplementary Tables

**Table S1: Hard- and software specifications of each fitness tracker model, related to STAR Methods.**<br>Precise description of the investigated devices with brand, type and firmware version.	
**Manufacturer with device name**	**Firmware**
Apple (Cuppertino, California, USA); Watch 7	watchOS8.1
FitBit (San Francisco, California, USA); Sense	5.3 (44.128.6.12)
Garmin (Olathe, Kansas, USA);Fenix 6 pro	19.20 (0fe794a)
Withings (Issy-les-Moulineaux, France); ScanWatch	2291

Table S2: Patient metadata, related to STAR Methods.
Metadata of the patients visualised as boxplots, including Median with Interquartile Range (IQR).

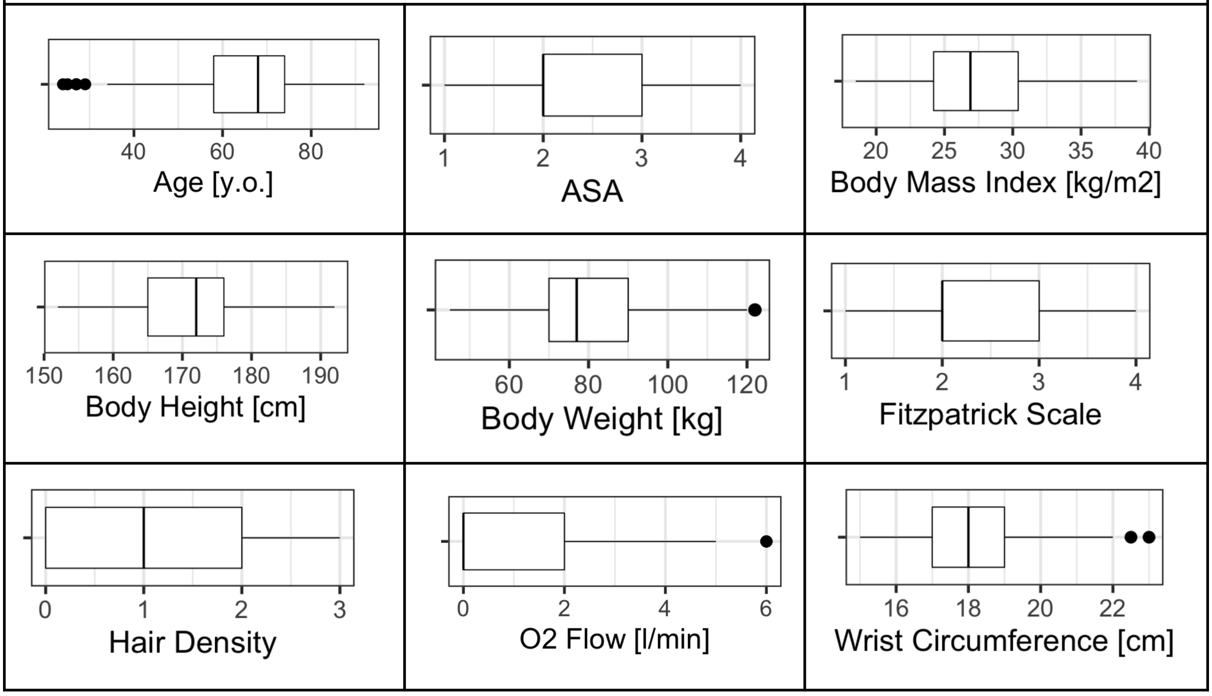

Table S3. Benchmarking indicators, related to Table 1.
Summary of the most important parameters for evaluating the measurement accuracy of the different manufacturers. The first row specifies the comparison against SaO_2, the second row against FO_2Hb and the third row against SpO_2.

Manufacturer	Philips	Apple	Garmin	Withings
m	109	84	93	57
	106	81	90	57
	–	253	272	169
Dropouts	0	25	15	49
	3	28	18	49
	–	81	55	163
Outlier	0	0	1	3
	0	0	1	3
	–	1	8	3
RMSE [CI]	2.20 [1.83;2.64]	2.44 [2.07;2.95]	3.50 [3.18;3.88]	2.32 [1.96;2.8]
	2.36 [2.11;2.68]	2.23 [1.99;2.23]	2.27 [1.99;2.62]	2.80 [2.31;3.42]
	–	2.60 [2.34;2.89]	3.36 [2.11;3.60]	3.43 [3.07;3.82]
MAE	1.58	1.90	3.07	1.88
	1.99	1.92	1.92	2.24
	–	1.89	2.71	2.64
MSE	4.85	5.97	12.24	5.37
	5.56	4.97	5.15	7.85
	–	6.74	11.30	11.74
MAPE	0.02	0.02	0.03	0.02
	0.02	0.02	0.02	0.02
	–	0.02	0.03	0.03
Bias [CI]	-1.00 [-1.38;-0.63]	-1.37 [-1.81;-0.93]	-2.73 [-3.18;-2.28]	-1.09 [-1.64;-0.54]

	1.27 [0.89;1.66]	0.89 [0.44;1.35]	-0.67 [-1.18;-0.16]	0.67 [-0.13;1.47]
	–	-0.52 [-0.84;-0.21]	-2.02 [-2.34;-1.70]	-0.7 [-1.21;-0.19]
Lower Limits of agreement [CI]	-4.94 [-5.59;-4.30]	-5.44 [-6.21;-4.68]	-7.13 [-7.91;-6.34]	-5.22 [-6.17;-4.27]
	-2.71 [-3.38;-2.05]	-3.22 [-4.01;-2.43]	-5.56 [-6.45;-4.68]	-5.36 [-6.74;-3.97]
	–	-5.62 [-6.16;-5.07]	-7.4 [-7.96;-6.85]	-7.43 [-8.31;-6.54]
Upper Limits of agreement [CI]	2.94 [2.29;3.59]	2.70 [1.94;3.47]	1.66 [0.88;2.45]	3.04 [2.09;3.98]
	5.26 [4.60;5.92]	5.00 [4.22;5.79]	4.23 [3.35;5.12]	6.70 [5.31;8.08]
	–	4.57 [4.03;5.12]	3.36 [2.80;3.92]	6.03 [5.15;6.92]
r	0.78	0.64	0.46	0.6
	0.77	0.65	0.45	0.45
	–	0.62	0.56	0.46
slope	1.2	0.83	0.59	0.64
	1.16	0.84	0.54	0.52
	–	0.59	0.54	0.43
shift	-20.49	15.17	37.26	34.33
	-14.4	16.11	43.03	46.11
	–	38.78	42.28	53.82
r_c	0.66	0.53	0.24	0.54
	0.63	0.59	0.43	0.52
	–	0.61	0.45	0.45
p-value	3.33^{-23}	3.71^{-11}	2.67^{-06}	7.26^{-7}
	3.98^{-22}	4.63^{-11}	9.38^{-06}	5.53^{-04}
	–	2.29^{-28}	2.88^{-24}	3.09^{-10}

m= measurement points. LOA = Limits of agreement. r= Pearson Correlation coefficient. r_c = Lin's concordance coefficient. RMSE = root-mean-square error. MAE = mean absolute error. MSE = mean of squared errors. MAPE = mean absolute percentage error.

Table S4. Effect of shivering on drop out rates, related to Figure 5.
Summary of the numerical results of the analysis of shivering on the dropout rates, separated for the investigated devices and between the different groups with corresponding p-values according to fisher-test.

	**Shivering Group**			**Reference Group**			**Fisher-test**
	Dropouts	*Values*	*%*	*Dropouts*	*Values*	*%*	*p-Value*
**TPO**	0	21	0	1	314	0.32%	1
**Apple**	11	10	52.38	71	244	22.54	0.006
**Garmin**	6	15	28.57	50	265	15.87	0.14
**Withings**	19	2	90.48	145	170	46.03	<0.001

TPO = transmissive pulse oximetry.
